# Optimization of glutathione production in *Saccharomyces cerevisiae* HBSD-W08 using Plackett–Burman and central composite rotatable designs

**DOI:** 10.1186/s12866-022-02742-4

**Published:** 2023-01-11

**Authors:** Xinyi Hu, Xinlei Shen, Shen Zhu, Huawei Zeng, Yuying Shuai

**Affiliations:** 1 College of pharmaceutical science, Zhejiang Chinese Medical University, Zhejiang, 310053 Hangzhou China; 2 Department of Bioengineering, College of Life Science, Huaibei Normal University, Huaibei, 235000 Anhui China

**Keywords:** GSH synthesis, Fermentation optimization, *Saccharomyces cerevisiae*, Plackett-Burman, Central composite rotatable design

## Abstract

**Background:**

Glutathione is an important bioactive tripeptide and is widely used in the food, medicine, and cosmetics industries. The aim of this study was to provide an efficient method for producing GSH and to explore its synthesis mechanism. *Saccharomyces cerevisiae* strain HBSD-W08 was screened for GSH production, and its fermentation medium was optimized using single-factor experiments of the Plackett-Burman and central composite rotatable designs. This method was used to analyze the effects of the presence and concentration of various carbon sources, organic and inorganic nitrogen sources, metal ions, and precursor amino acids on GSH production and catalase, superoxide dismutase, and γ-glutamylcysteine synthetase activity.

**Results:**

The three most significant factors affecting GSH production were peptone (optimal concentration [OC]: 2.50 g L^− 1^), KH_2_PO_4_ (OC: 0.13 g L^− 1^), and glutamic acid (OC: 0.10 g L^− 1^). GSH productivity of HBSD-W08 was obtained at 3.70 g L^− 1^ in the optimized medium. The activity of γ-GCS, which is a marker for oxidative stress, was found to be highly positively correlated with GSH production.

**Conclusions:**

This finding revealed an underlying relationship between GSH synthesis and oxidative stress, providing useful information for developing effective GSH fermentation control strategies.

## Background

Glutathione (GSH) is a bioactive tripeptide composed of glutamic acid, cysteine, and glycine that forms compounds with γ-glutamyl and active sulfhydryl groups and has important physiological functions in animals, plants, and microorganisms [1, 2]. GSH participates in various biochemical reactions in vivo and is known for its antioxidant, anti-allergy, and detoxification properties. Glutathione-*S*-transferases (GSTs) catalyze GSH conjugation with drugs and xenobiotics. GSH is a key factor in redox signaling through its participation in trans hydrogenation reactions that remove hydrogen peroxide, free radicals, and other peroxides. These signaling pathways also play important roles in regulating cell proliferation, apoptosis, and immune function. In addition, decreased GSH concentration may be related to the pathogenesis of many diseases, including rheumatoid arthritis and Alzheimer’s disease [3, 4]. Thus, GSH is recommended as a therapeutic agent by the food, pharmaceutical, and cosmetic industries [5].

GSH production via chemical, enzymatic, and fermentation processes has been studied extensively [6–8]. Of these, the liquid fermentation method is the most cost-effective in terms of raw materials required to synthesize GSH using specific microbial metabolism; it is considered a scalable, affordable, simple, and efficient production method. The most common strategy is the addition of amino acids such as cysteine to the medium [9, 10]. Some studies have used specific strains with increased activity of key enzymes for GSH synthesis [11]. Others have tried the addition of various substances (i.e., mannans, peptones, hydrolyzed extracts, ATP, and sodium dodecyl sulfate) to increase industrial yield potential [10, 12–14]. In addition, other factors affecting the production of GSH have been studied. Liang et al. adopted a three-stage operation (batch fermentation, exponential glucose feeding, and constant glucose feeding) to improve GSH yield and studied the effect of dissolved oxygen concentration on cysteine oxidation and GSH yield [15]. Although the physiological mechanism of GSH synthesis is well studied, research on the underlying mechanisms of liquid fermentation is limited. GSH is synthesized through a two-step ATP-dependent pathway consisting of γ-glutamylcysteine synthase (γ-GCS) and glutathione synthetase (GS) in organisms. The effects of the relavant enzymes in *S. cerevisiae* cells and different carbon sources on the intracellular protein content need to be determined, and their any associations with GSH production need to be identified.

The objectives of this study were to screen different isolates of *S. cerevisiae* for GSH production and then use the single factor optimization method, Plackett-Burman central composite rotatable design (P-B CCRD), and response surface methodology (RSM) to optimize fermentation conditions for maximal GSH production. In addition, the mechanism of GSH synthesis was preliminarily explored to provide a further basis for large-scale production.

## Methods

### Strain screening

Thirteen *S. cerevisiae* strains (HBSD-W01–013) previously isolated from soil samples and preserved in Huaibei Normal University were inoculated on a YEPD solid medium containing 10 g L^− 1^ yeast extract, 20 g L^− 1^ peptone, 20 g L^− 1^ glucose, and 20 g L^− 1^ agar (pH = 6.0) for 48 h at 30 °C. Then, the seeds were transferred into a fermentation medium, containing 30 g L^− 1^ glucose, 20 g L^− 1^ peptone, 0.9 g L^− 1^ KH_2_PO_4_, 10 g L^− 1^ MgSO_4_, and 5 g L^− 1^ (NH_4_)_2_SO_4_, and cultivated in a rotary shaker (HZQ-2, Bossesway, China) (200 r min^− 1^) for 48 h at 30 °C. The isolates were screened for intracellular GSH production using cell lysates. The strains showing the highest GSH production were selected for further research and characterized based on 16S rDNA sequence analysis conducted at Kechang Biotechnology Co., Ltd. (Nanchang, Jiangxi, China).

### Determination of *S. cerevisiae* biomass and GSH production

Cells were obtained from the medium by centrifugation (Avanti J-26XP, Beckman Coulter, USA) at 10,000×*g* and 4 °C for 10 min. Next, the pellet was washed twice with 65 mM phosphate-buffered saline (PBS, pH = 7.8) and dried at 85 °C to a constant weight. The dry weight of cells per liter of culture medium was determined.

After centrifugation, the pellets were resuspended in PBS, and the cell suspension was disrupted using an ultra-sonicator (Q125, Q-sonica, USA) in an ice bath for 15 min. Cellular debris was removed by centrifugation at 10,000×g and 4 °C for 10 min. Next, cell lysates were used to quantify GSH levels.

GSH production was measured using the 5,5′-dithio-bis (2-nitrobenzoic acid) (DTNB) method. Briefly, 3 mL of 0.15 M NaOH and 1 mL of 3% formaldehyde were added to 2 mL of the cell disruption supernatant and mixed well. Next, 5 mL of 0.1 mM DTNB analytical reagent (DTNB solution with Tris/HCl buffer) was added, and the mixture was incubated at 25 °C for 15 min. GSH was quantified by spectrophotometry at 412 nm. Each measured variable represented results obtained from three separate experiments.

### Optimization of GSH production and Plackett–Burman and central composite rotatable designs

The single-factor optimization method was used to analyze different carbon sources (fructose, lactose, citric acid, xylose, maltose, glucose, glycerin, dextrin, and sucrose), organic nitrogen sources (skim milk, yeast powder, beef extract, and peptone), inorganic nitrogen sources (NH_4_Cl, (NH_4_)_2_SO_4_, NaNO_3_, and org-N), metal ions (Mg^2+^ and K^+^), precursor amino acids (glutamic acid, cysteine, and glycine) as well as other factors and to identify the most effective substances for GSH production.

The Plackett-Burman (P-B) experimental design was used to evaluate the significance of multiple variables in medium compositions in GSH production. Six factors, including glucose, peptone, (NH_4_)_2_SO_4_, MgSO_4_, potassium dihydrogen phosphate (KH_2_PO_4_), and glutamic acid, were used to evaluate GSH production, whereas other factors were used at optimal concentration (OC) as determined by the single-factor optimization experiments. The factors and levels of P-B are presented in Table 1. Six parameters were tested at two levels. In total, 12 experiments were conducted to study the six selected parameters. P-B does not describe the interaction between any factors but is used to determine the important factors influencing GSH production. Based on the results of P-B, a Central Composite Rotatable Design (CCRD) was conducted with three levels and three variables (Table 2).

**Table 1:** Factors and levels of Plackett-Burman design

Number	Factor	Level		
		− 1	0	+ 1
A	Glucose	3%	4%	5%
B	Peptone	3%	4%	5%
C	KH_2_PO_4_	0.25	0.5	0.75
D	Magnesium Sulfate	0.5%	1%	1.5%
E	Ammonium Sulfate	0.75%	1.0%	1.5%
F	Glutamate	0.1%	0.15%	0.2%

**Table 2:** Concentration ranges of the variables used in the central composite design

Factor	Level		
	−1	0	+ 1
KH_2_PO_4_	0.25	0.5	0.75
Glutamate	0.1%	0.15%	0.2%
Peptone	3%	4%	5%

Fermentation conditions were optimized using three factors selected by P-B CCRD with a three-level RSM. One factor was fixed at a level of zero, whereas the function of the other two factors was used to obtain the quadratic regression equation.

### Statistical analyses

Statistical analysis and curved surface diagram were performed using Minitab 19 (Minitab, State College, PA, USA). The experimental data of the single-factor optimization method were plotted using GraphPad Prism 8.

### The effect of different carbon sources on intracellular protein content and activity of catalase (CAT), superoxide dismutase (SOD), and γ-glutamylcysteine synthetase (γ-GCS) in *S. cerevisiae* cells and statistical analysis of the data

To explore the underlying mechanism of GSH synthesis, we aimed to determine the effect of different carbon sources on the intracellular protein content and the activity of CAT, SOD and γ-GCS in *S. cerevisiae* cells and identify any associations with GSH production.

Determination of intracellular protein was carried out according to a previously established method: Coomassie brilliant blue was used in this experiment, and bovine serum albumin was used as the standard [16].

Determination of intracellular CAT: 1 mL of the crude enzyme solution sample, 1 mL of 50 mmol/L NaHPO_4_ -- NaH_2_PO_4_ buffer (pH = 7.0), and 1 mL of 120 mmol/L H_2_O_2_ were mixed with each other, and the OD value was measured at 240 nm after the reaction was complete. Three groups of parallel measurements were set up, while one group was set as the control group with the boiled enzyme solution as the sample. The activity of CAT was calulated to reduce the enzyme amount by 0.1 in 1 mL to one enzyme activity unites (A_240_).

Determination of SOD: A modified procedure of pyrogallol autoxidation was used in this experiment and the absorbance of fluid under test was measured at 325 nm at the initial time and 1 min later [17]. The results were expressed as the amount of enzyme required to inhibit pyrogallol autooxidation by 50%.

Determination of γ-GCS activity: A 3 mL reaction system was prepared, including 0.1 mL of the crude enzyme solution sample, 100 mmol/L Tris-HCl with 20 mmol/L sodium glutamate, 10 mmol/L cysteine, 5 mmol/L adenosine triphosphate disodium, 100 mmol/L NaCl, 20 mmol/L magnesium chloride, and an appropriate amount of crude enzyme solution. The reaction system was placed in a 37 °C water bath for 30 min, and finally, 0.3 mL 10% trichloroacetic acid was added to terminate the reaction. The supernatant after centrifugation was taken and an appropriate amount of 5 mol/L sulfuric acid and 20% ammonium molybdate was added for color development. The absorbance was measured at the maximum absorption peak of 636 nm. The activity of γ-GCS was calculated by the regression equation of the standard curve. The amount of enzyme required to produce 1 μmol of inorganic phosphorus per mg of protein ATP consumption was defined as an ATPase activity unit.

GSH content was taken as the vertical axis, and the ratio of each index (protein concentration, CAT yield, SOD yield, and γ-GCS yield) to biomass was taken as the abscissa. The trend chart was made, and the correlation was judged according to R^2^.

## Results and discussion

### Screening of *S. cerevisiae* strains

Thirteen *S. cerevisiae* strains from Huaibei Normal University were screened for GSH production activity, five were selected for their GSH production (Table 3). As shown in Table 3, HBSD-W08 did not have the highest biomass but showed significantly higher GSH production (0.88 g L^− 1^), relatively close to the GSH-producing activity of 0.899 g L^− 1^ (3.44 g L^− 1^ after optimization) exhibited by waste brewer’s yeast and commercial baker’s yeast [10]. Thus, HBSD-W08 was selected for studying the effects of culture conditions on GSH production. The strain HBSD-W08 was identified as *S. cerevisiae* by Kechang Biotechnology Co., Ltd. (Nanchang, Jiangxi, China).

**Table 3:** Five *S. cerevisiae* strains with relatively higher GSH production

Strains	Biomass (g/L)	GSH (g/g%)*	GSH production (g/L)
HBSD-W02	9.3 ± 0.07	1.1 ± 0.06	0.1 ± 0.05
HBSD-W03	6.5 ± 0.06	1.8 ± 0.07	0.12 ± 0.04
HBSD-W08	16.7 ± 0.05	5.3 ± 0.05	0.88 ± 0.04
HBSD-W15	5.8 ± 0.06	1.6 ± 0.05	0.09 ± 0.03
HBSD-W19	18.6 ± 0.05	2.0 ± 0.07	0.37 ± 0.04

1. Data were expressed as mean ± SD from three independent experiments

2. GSH (g/g%)*:g/g% means the content of GSH / the content of biomass%

### Single factor experiments to investigate the effect of medium composition on GSH yield

#### Effect of carbon source and concentration

**Fig. 1:**
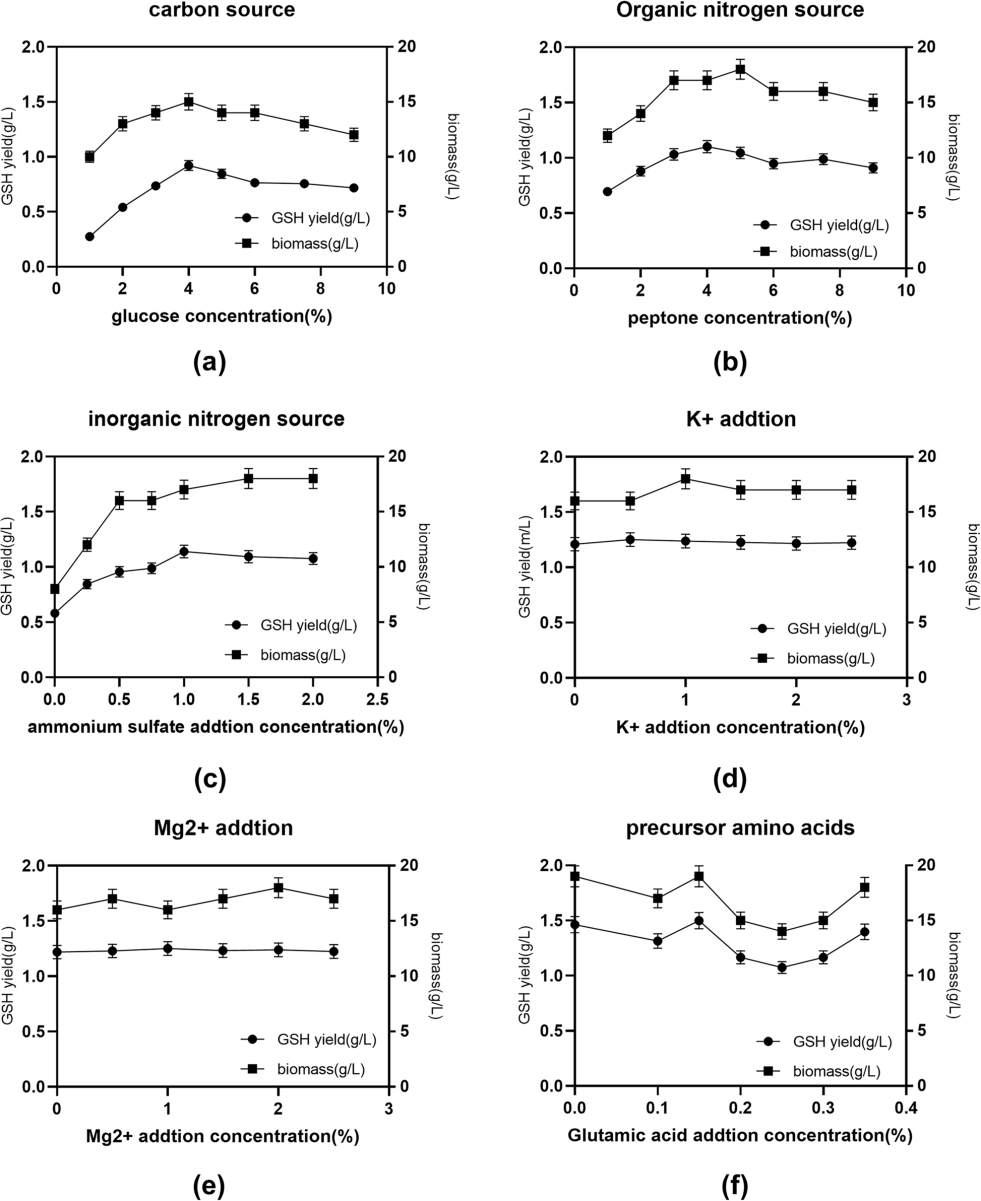
Single factor test results. **a** The effects of different glucose concentrations on GSH yield and biomass. **b** The effects of different peptone concentrations on GSH yield and biomass. **c** The effects of different ammonium sulfate concentrations on GSH yield and biomass. **d** The effects of different K^+^ addtion concentrations on GSH yield and biomass. **e** The effects of different Mg^2+^ addtion concentrations on GSH yield and biomass. **f** The effects of different glutamic acid addtion concentrations on GSH yield and biomass

Of the nine different carbon sources that were studied, glucose had the most positive effect on HBSD-W08 biomass and GSH production, results that were in accordance with those presented in previously published studies [18]. The optimal glucose concentration in the fermentation medium was 4%, at which both the HBSD-W08 biomass (15 g L^− 1^) and GSH production (0.92 g L^− 1^) were the highest (Fig. 1(a)). Both variables were decreased at glucose concentrations higher than 4%, probably because the cell growth was inhibited by high osmotic pressure [19, 20]. Thus, glucose was used as a carbon source for P-B CCRD.

#### Effect of organic nitrogen source and concentration

The effects of different organic nitrogen sources on HBSD-W08 biomass and GSH production were investigated. HBSD-W08 biomass and GSH production were the highest when peptone was used as an organic nitrogen source, whereas they were the lowest when skim milk was used. The results were consistent with those reported in previously published studies [18, 21]. As shown in Fig. 1(b), the OC of peptone was 4%, at which GSH production reached a maximum of 1.1 g L^− 1^. Any increase in concentration beyond 4% led to a decrease in HBSD-W08 biomass and GSH production. Therefore, peptone was used as an organic nitrogen source for P-B CCRD.

#### Selection of inorganic nitrogen source and concentration

Some studies showed that inorganic nitrogen significantly improved *S. cerevisiae* biomass and GSH production [21]. Our results suggested that, compared with other inorganic nitrogen sources, (NH_4_)_2_SO_4_ had the most positive effect on both variables. Its OC was determined to be 1.0% (Fig. 1(c)) and GSH production was as high as 1.14 g L^− 1^. Previous studies suggested that (NH_4_)_2_SO_4_ helps yeast cells to maintain a relatively high intracellular GSH level [22]. Thus, (NH_4_)_2_SO_4_ was used as an inorganic nitrogen source for P-B CCRD.

#### Selection of metal ion and concentration

The addition of different metal ions (such as Mg^2+^, K^+^, etc) can promote the production of GSH by *S. cerevisiae* [23]. As shown in Fig. 1(d) and (e), HBSD-W08 biomass and GSH production peaked at 1.25 g L^− 1^ with the addition of 1% Mg^2+^ and 0.5% K^+^; however, any further increase in ion concentration had a negative effect on both variables, probably due to the high osmotic pressure that hindered the cells metabolic activity. Past studies reported that genes encoding sodium/proton antiporter and ATPase help yeast cells to express heterologous alkali-metal-cation exporters and improve their tolerance to metal ions and salinity [24]. Thus, both Mg^2+^ and K^+^ were used as metal ions for P-B CCRD.

#### Selection of precursor amino acids and concentrations

Of the three different precursor amino acids that were studied, glutamic acid had the most positive effect on HBSD-W08 biomass and GSH production, results that were in accordance with those presented in previously published studies [25]. As shown in Fig. 1(f), the addition of glutamic acid showed inconsistent effects on GSH production, however, it reached a peak value of 1.5 g L^− 1^ when the glutamic acid concentration was 0.15%. Previous studies also increased GSH production when a combination of cysteine, glutamic acid, and glycine was used in yeast bioconversion media [26]. Thus, glutamic acid was used as an amino acid for P-B CCRD.

### P-B CCRD and screening of factors affecting GSH production

A P-B design with *N* = 12 was selected, and GSH yield (g L^− 1^) in liquid fermentation medium was used as the response value. Experimental design and results (mean value for three repetitions) are shown in Table 4, and the analysis of variable are shown in Table 5 and ANOVA results are shown in Table 6.

**Table 4:** P-B Experimental Design and results

NO.	A	B	C	D	E	F	GSH yield(g L^− 1^)
1	1	−1	1	−1	1	−1	3.2029
2	−1	1	1	1	−1	1	2.4931
3	−1	− 1	−1	1	1	1	0.6662
4	1	−1	−1	-1	-1	1	1.0967
5	-1	-1	-1	-1	-1	-1	1.3644
6	1	1	-1	1	-1	-1	2.5629
7	-1	1	-1	-1	1	1	2.1905
8	-1	1	1	-1	-1	-1	3.4822
9	-1	-1	1	1	1	-1	2.4233
10	1	1	1	-1	1	1	3.3775
11	1	1	-1	1	1	-1	2.2255
12	1	-1	1	1	-1	1	2.4931

**Table 5:** Analysis of variable for P-B design

Variable	Effect	Coefficients	Standard error	*T* value	*P* value
Glucose	0.3898	0.1949	0.0789	2.47	0.057
Peptone	0.8475	0.4238	0.0789	5.37	0.003
KH_2_PO_4_	1.2277	0.6138	0.0789	7.78	0.001
MgSO_4_	−0.3084	−0.1524	0.0789	−1.95	0.108
(NH_4_)_2_SO_4_	0.0989	0.0495	0.0789	0.63	0.558
Glutamate	−0.4907	− 0.2453	0.0789	−3.11	0.027
R-Sq		Predicted R-Sq		Adjusted R-Sq	
95.62%		74.79%		90.37%

**Table 6:** ANOVA of P-B design

Source	DOF	Adj SS	Adj MS	*F* value	*P* value
model	6	8.16900	1.36150	18.21	0.003
A	1	0.45587	0.45587	6.10	0.057
B	1	2.15485	2.15485	28.82	0.003
C	1	4.52137	4.52137	60.48	0.001
D	1	0.28524	0.28524	3.82	0.108
E	1	0.02935	0.02935	0.39	0.558
F	1	0.72231	0.72231	9.66	0.027
Error	5	0.37381	0.07476		

The regression equation is Production = 2.2982 + 0.1949 A + 0.4238 B + 0.6138 C - 0.1542 D + 0.0495 E - 0.2453 F. Regression equation *R*^2^ = 95.62%, indicating that 95.62% of the data can be explained by the model, which fits well with the actual situation. Our results showed that the influence of factors on the GSH yield is KH_2_PO_4_ > peptone > glutamate, among which the influence of KH_2_PO_4_ and peptone is extremely significant (*P* < 0.01), and the influence of glutamate is significant (*P* < 0.05). The other factors were not significant (*P* > 0.05). The *P* value of the model (*P* < 0.01) indicates that the equation model is very significant.

Experimental design and results of the central combination experiment design (repeated for 3 times and averaged) are shown in Table 7.

**Table 7:** Central Composite Test Design and results

Number	Peptone/(g L^− 1^)	KH_2_PO_4_/(g L^− 1^)	Glutamic acid/(g L^− 1^)	GSH yield/(g L^− 1^)
1	2.00	0.000	0.075	3.6186
2	2.00	0.545	0.075	3.4088
3	2.00	0.250	0.000	2.1884
4	2.50	0.250	0.151	1.1587
5	2.50	0.125	0.100	3.7330
6	2.50	0.375	0.050	3.1037
7	2.50	0.375	0.100	3.5614
8	2.50	0.125	0.050	3.4279
9	1.50	0.375	0.100	2.9703
10	1.50	0.375	0.050	3.1991
11	0.14	0.250	0.075	1.7880
12	3.86	0.250	0.075	2.9893
13	1.50	0.125	0.050	3.3326
14	1.50	0.125	0.100	3.4470
15	2.00	0.250	0.075	3.6681

R-Square = 93.19% Adjusted R-Square = 80.95%

**Fig. 2:**
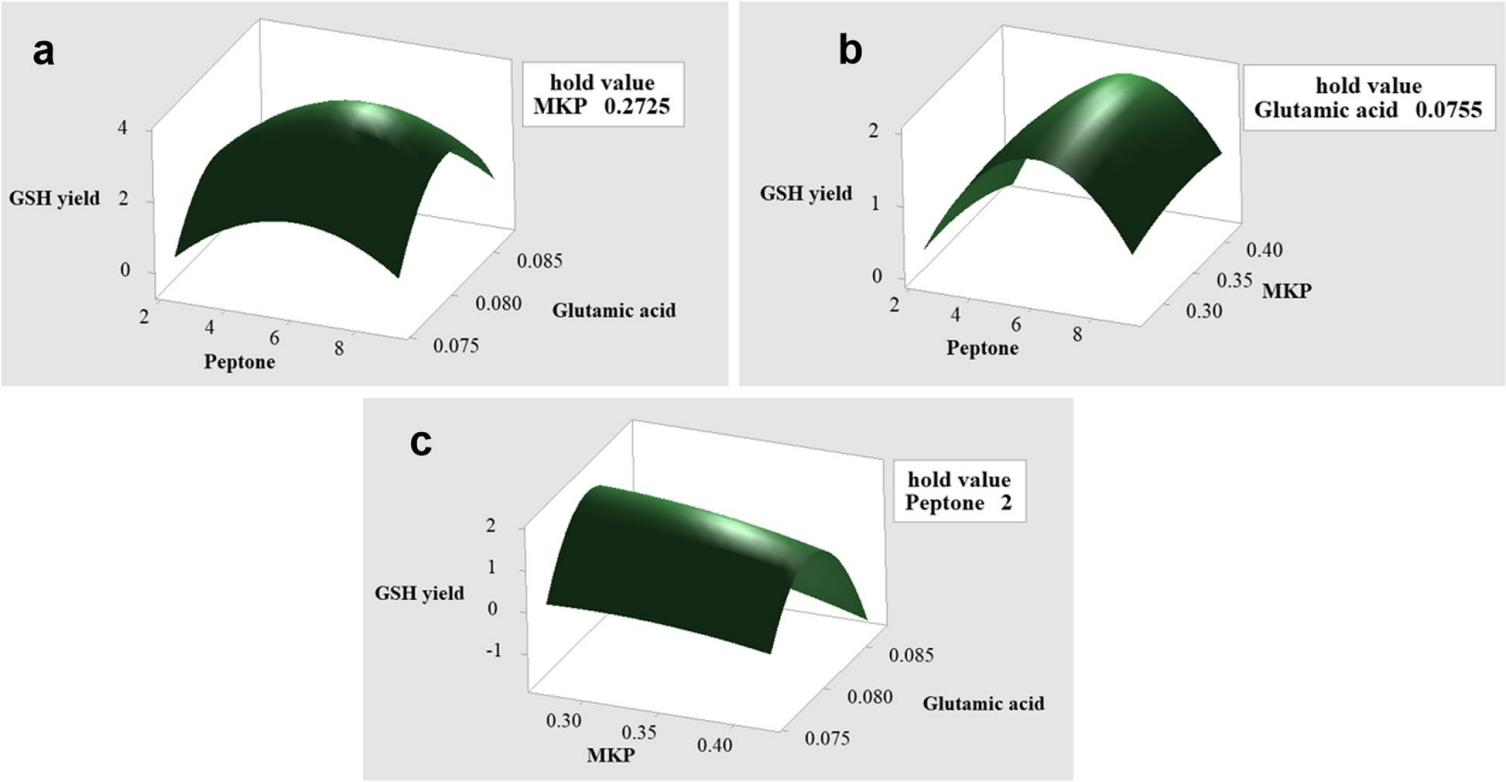
Curved surface diagram of effects of the interaction of three chemical substances on GSH yield. **a** The effect of the interaction of peptone and glutamic acid on the production of GSH. **b** The effect of the interaction of KH_2_PO_4_ and peptone on the production of GSH. **c** The effect of the interaction of KH_2_PO_4_ and glutamic acid on the production of GSH

The three-dimensional map of the response surface was constructed using Minitab 19, which showed the effects of the interaction of peptone, KH_2_PO_4_ and glutamic aicd on the total production of GSH (Fig. 2). When a certain factor is fixed the production of GSH increases rapidly with the increase of the other two factors and then decreases after reaching a peak. This shows that the appropriate control of peptone, KH_2_PO_4_, and glutamic acid is beneficial to improving the total production of GSH. The composition of the optimal medium was peptone 2.50 g L^− 1^, KH_2_PO_4_ 0.13 g L^− 1^, and glutamic acid 0.10 g L^− 1^ where the predicted maximum yield of GSH was 3.73 g L^− 1^. The experiment was repeated 5 times, and the average value was 3.67 g L^− 1^ which indicated that the model was effective in predicting the production of GSH.

### Optimization of fermentation conditions

The interaction of peptone (OC: 2.50 g L^− 1^), KH_2_PO_4_ (OC: 0.13 g L^− 1^), and glutamic acid (OC: 0.10 g L^− 1^) significantly increased GSH production at 3.70 g L^− 1^. Li et al. adopted a two-step reaction in which yeast cells first synthesized only γ-GCS and they then added glycine to facilitate GSH synthesis [10]. The maximum yield of GSH using this method was 3.44 g L^− 1^ within 30 h.

### GSH production mechanism

Traditionally, GSH is synthesized through a two-step ATP-dependent pathway consisting of γ-GCS and GS in organisms. γ-GCS is one of the rate-limiting enzymes for the synthesis of GSH, so the activity of γ-GCS directly affects the yield of GSH. SOD and CAT can scavenge oxygen free radicals, and are GSH-related antioxidant enzymes in organisms, so it was necessary to explore the relationship between SOD, CAT and GSH production. Thus the activity of CAT, SOD, γ-GCS and also the intracellular protein were detected.

**Fig. 3:**
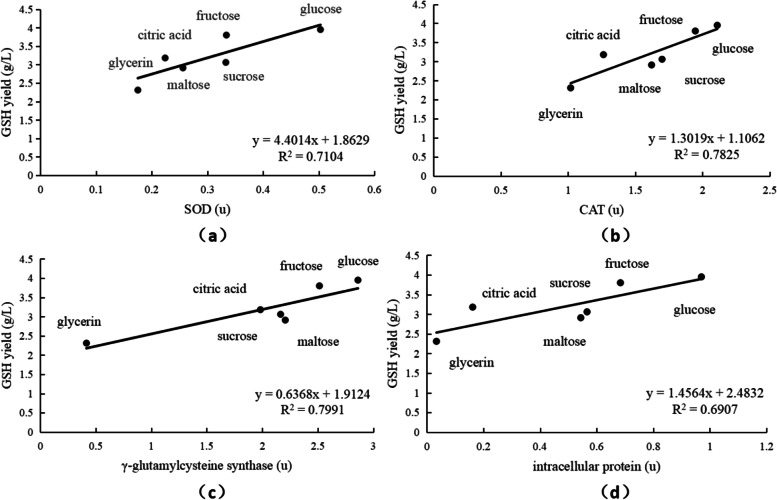
Diagram of the relationship between four measured indicators of different carbon sources and GSH yield. **a** The effect of different carbon sources on activity of SOD in *S. cerevisiae* strains. **b** The effect of different carbon sources on activity of CAT in *S. cerevisiae* strains. **c** The effect of different carbon sources on activity of γ-GCS in *S. cerevisiae* strains. **d** The effect of different carbon sources on intracellular protein content in *S. cerevisiae* strains

It can be seen from Fig. 3 that the measured indicators (intracellular protein, CAT, SOD, γ-GCS activity) are correlated with the production of GSH. The correlation size was: γ-GCS > CAT > SOD > intracellular protein.

As shown in Fig. 3, all parameters were positively correlated with GSH production. In particular, the activity of γ-GCS showed a strong correlation (*r* = 0.8939) with GSH production, suggesting that oxidative stress might promote the expression of GSH-related synthetic genes. GSH is synthesized by γ-GCS and GS. γ-GCS is the cause of the special peptide bond of GS. It first connects the γ-carboxyl group of glutamic acid to the amino group of cysteine to form the precursor of GSH-γ-glutamylcysteine. Then, GS connects glycine to it to produce GSH. γ-GCS and GS are encoded by genes GSHl and GSH2, respectively. Previous studies have shown that the up-regulation of genes GSHl can increase GSH production, but the up-regulation of GSH2 has no obvious change [27]. Therefore, the expression of GSHl directly restricts the production of GSH. Fan [28] introduced the plasmid containing the GCS-I gene into yeast cells, and the GSH content increased by 0.5 times compared with the original strain, which also showed the correlation between GSH expression and GCS. Thus, an increase in γ-GCS might be conducive to GSH production [29, 30].

## Conclusion

In the present study, *S. cerevisiae* HBSD-W08 was selected for its high GSH production and used for developing an optimal fermentation medium using the single-factor optimization method, P-B CCRD, and RSM. The results showed that peptone, KH_2_PO_4_ and glutamate were the three most significant factors affecting the GSH production of *S. cerevisiae* HSD-W08. The optimum concentration for GSH production of peptone was 2.50 g L^− 1^, KH_2_PO_4_ was 0.13 g L^− 1^ and glutamate was 0.10 g L^− 1^. The highest GSH yield was 3.70 g L^− 1^. Furthermore, the underlying mechanism of GSH production was explored. The activity of γ-GCS, which is a marker for oxidative stress, was found to be highly positively correlated with GSH production. This finding revealed an underlying relationship between GSH synthesis and oxidative stress, providing useful information for developing effective GSH fermentation control strategies.

## Data Availability

The datasets used and/or analyzed during the current study are available from the corresponding author on reasonable request.

## Abbreviations

GSH: Glutathione
OC: optimal concentration
DTNB: 5,5′-dithio-bis (2-nitrobenzoic acid)
P-B CCRD: Plackett-Burman central composite rotatable design
CAT: catalase
SOD: superoxide dismutase
GSTs: Glutathione-S-transferases
γ-GCS: γ-glutamylcysteine synthetase
GS: glutathione synthetase
RSM: response surface methodology
